# The Release of the Bromodomain Ligand *N,N*-Dimethylacetamide Adds Bioactivity to a Resorbable Guided Bone Regeneration Membrane in a Rabbit Calvarial Defect Model

**DOI:** 10.3390/ma13030501

**Published:** 2020-01-21

**Authors:** Barbara Siegenthaler, Chafik Ghayor, Nisarat Ruangsawasdi, Franz E. Weber

**Affiliations:** 1 Oral Biotechnology & Bioengineering, Center for Dental Medicine/MKG, University of Zürich, 8032 Zurich, Switzerland; barbara.m.siegenthaler@gmail.com (B.S.); chafik.ghayor@usz.ch (C.G.); 2 Department of Pharmacology, Faculty of Dentistry, Mahidol University, 10400 Bangkok, Thailand; nisarat_mac@outlook.co.th; 3 Zurich Center for Integrative Human Physiology (ZIHP), University of Zurich, 8057 Zurich, Switzerland; 4 CABMM Center for Applied Biotechnology and Molecular Medicine, University of Zurich, 8057 Zurich, Switzerland

**Keywords:** GBR, guided bone regeneration, *N,N*-Dimethylacetamide, DMA, bioactive membrane

## Abstract

*N,N*-Dimethylacetamide (DMA) is FDA approved as an excipient and is used as drug-delivery vehicle. Due to its amphipathic nature and diverse bioactivities, it appears to be a good combination of biodegradable poly-lactide-co-glycolide (PLGA)-based guided bone regeneration membranes. Here we show that the solvent DMA can be loaded to PLGA membranes by different regimes, leading to distinct release profiles, and enhancing the bone regeneration in vivo. Our results highlight the potential therapeutic benefits of DMA in guided bone regeneration procedures, in combination with biodegradable PLGA membranes.

## 1. Introduction

Guided bone regeneration (GBR) is a procedure used frequently in dentistry to restore alveolar deficiencies and to prepare the site for the placement of dental implants [1]. The rationale for applying a membrane is to prevent the ingrowth of non-osteogenic tissues into the space where bone formation should occur [2]. The membrane can be applied with or without bone substitute materials [3,4]. In the first case, the membrane, in addition to installing compartmentation, prevents granular bone substitutes from dispersion. Since the time when guided bone regeneration by membranes was established, a multitude of materials were tested [5,6,7]. Originally, the non-resorbable expanded polytetrafluorethylen (ePTFE) membranes, and more recently the resorbable native collagen membranes, are used most frequently [8].

The switch from non-resorbable membranes to resorbable membranes changes the clinical procedure substantially, since it obviates the need for a second surgery to remove the non-resorbable material. This feature also distinguishes the first generation, non-resorbable from the resorbable second generation membrane [9]. The third generation of GBR membranes that are not only occlusive and resorbable, but also exert bioactivity to biologically stimulate osteoprogenitor cells for enhanced bone growth, was achieved more than a decade ago by the combination of a poly-lactide-co-glycolide (PLGA) membrane with the plasticizer *N*-methyl-pyrrolidone (NMP) [10]. From the broad spectrum of NMP’s bioactivities, NMP released by the membrane appeared attractive for guided bone regeneration. NMP’s bioactivities encompass the enhancement of the activity of bone morphogenetic proteins (BMPs) [11], inhibition of osteoclast formation and activity [12], facilitation of bone regeneration in inflamed states [13,14], and reduced bone loss under osteoporosis [15]. Despite all these advantageous activities, the performance of the NMP-releasing PLGA membrane in clinical trials was not superior when compared to the more established resorbable and non-resorbable membranes [16,17]. More recent work on the applications of NMP and BMP for sinus lifts yielded inconsistent effects on bone regeneration, resulting in no significant benefits compared to controls [18]. Taken together, the usage of the organic solvent NMP as a drug delivered by bone substitutes or GBR membranes failed so far to be translated into the clinic.

Organic solvents are commonly used in the pharmaceutical industry as reaction media, for product synthesis, and as drug vehicles. *N*,*N*-Dimethylacetamide (DMA) is FDA approved as an excipient and therefore widely used as a drug-delivery vehicle [19]. In high-risk neuroblastoma treatment, for example, DMA, at a high concentration, is administered to facilitate chemotherapy by busulfan [20]. In 2013, Reznik and co-workers found DMA to be bioactive [21]. We showed that DMA binds bromodomains, and is thus epigenetically active in bone regeneration, bone degradation, and osteoporosis [22]. In conjunction with guided bone-regeneration procedures, its ability to enhance bone regeneration and to inhibit bone destruction by osteoclasts [22] could be beneficial.

Here we report how a PLGA-based membrane can be loaded with DMA, and how the loading regime affects the surface of the membrane and the release of DMA. Moreover, we performed a preclinical test of a DMA-loaded PLGA membrane in a GBR membrane model in the rabbit calvaria.

## 2. Materials and Methods

### 2.1. Chemical Loading of PLGA Membranes

*N*,*N*-Dimethylacetamide (DMA) anhydrous with 99.8% purity (Sigma-Aldrich Chemie GmbH, St. Gallen, Switzerland) was used for the experiments. PLGA membranes (Inion GTRTM membranes, Inion Oy, Tampere, Finland, 30 × 40 mm; 0.2 mm thick) were loaded with pure DMA by two different techniques: (1) a vapor deposition method and (2) direct dipping into the liquid chemical. Membranes were loaded to reach a weight gain of 10%, 25%, and 50%; hereafter named vapor 10%, vapor 25%, and vapor 50%. Vapor deposition was performed at room temperature in a desiccator connected to a vacuum pump. Inside the desiccator, the membranes were placed onto a metal mesh laying over a glass dish filled with pure DMA. To assess the weight gain of the membranes during the loading protocol, membranes were weighed before, during, and after vapor deposition on a chemical balance, and vapor deposition was allowed until intended loading percentage was reached. For 50% loading, the procedure took 22 h. Dip loading of the membrane to 50% was achieved by dipping the PLGA membrane into pure DMA for 10 s.

### 2.2. In Vitro Release of Chemicals

The release experiment was performed in triplicate. Equal-sized, chemical-loaded membrane samples were placed into glass bottles containing 50 mL of phosphate-buffered saline (PBS, pH 7.4). For the time course of the in vitro release, samples were kept agitating at 37 °C. At indicated time points (2 min up to 16 weeks), 200 µL of sample was removed and stored at 4 °C for subsequent analysis. DMA concentration was measured at 220 nm, using 96-well plates coated to enable reading at UV wavelengths (Corning, Corning, NY, USA). Values were compared to a standard curve of known DMA concentrations. To estimate the non-toxic concentration of the released DMA for the in vivo experiments, the membrane surface and release volume were taken into consideration.

### 2.3. Scanning Electron Microscopy for Structural Analysis

Chemical-loaded membranes were air-dried, fixed onto metal stubs, and gold-coated using a gold sputter machine (SCD 030, Baltec, Balzers, Lichtenstein). Parameters applied for sputtering and leading to a gold layer thickness of about 10 nm were the following: sputtering time 90 s, current 45 mA, and distance of gold target to sample 60 mm. The membrane structure was analyzed using the Zeiss Supra V50 SEM (Carl Zeiss, Oberkochen, Germany) at an acceleration voltage of 5 kV. Access to the SEM was kindly provided by the Center for Microscopy and Image Analysis (ZMB) at the University of Zürich.

### 2.4. Animal Model for Guided Bone Regeneration

All animal experiments were approved by the local authorities (Veterinäramt Zurich, Switzerland) under the licenses: 108/2012 and 115/2015. Six New Zealand white rabbits were used in this experiment. To initiate the operation, the animals were anesthetized by an injection of 65 mg/kg ketamine and 4 mg/kg xylazine, and maintained under anesthesia with isoflurane/O2. Opening of the soft tissue was followed by removal of the periosteum. Four defects of 6 mm in diameter were marked on each animal’s skull halfway into the cranial bone, using a trephine fixed in a dental hand piece. Defect areas were kept clean from bone debris by constant flushing with 0.9% saline solution. The creation of defects was completed using a round burr. For the guided bone regeneration method, one piece of the membrane, 7 mm in diameter, was pushed inside the defect and fixed in place by the intrinsic pressure of the brain and the dura mater. The second piece was placed on top of the defect, fixed by two titanium pins to allow for an empty space between the two membranes where the bone could regenerate in a guided manner as reported earlier [23]. One of the four defects was kept empty to serve as a control for non-treated bone regeneration and was termed empty control. A second defect was closed with untreated PLGA membranes not loaded with DMA, and one defect was treated with vapor 10% DMA membranes. The fourth defect was treated with a membrane loaded with an exploratory substance, which is not part of this study. The treatment modalities of the four defects were assigned randomly for the first animal. For the consecutive animals, the modalities were rotated clockwise by one defect. After treating the defects, soft tissues were closed by sutures and animals received analgesia specified in the license. Four weeks after surgery, the animals were sedated using barbiturates and an overdose of ketamine for sacrifice. Calvarial bones were excised and immediately processed for histology.

### 2.5. Histology

Excised calvarial bone samples were fixed with 70% ethanol, and dehydrated using a sequential water substitution process at 24 h in 40% ethanol, 72 h in 70% ethanol (solution replaced every 24 h), 72 h in 96% ethanol and a final dehydration step for 72 h in 100% ethanol (solution replaced every 24 h). Fixation and defatting were allowed for 72 h in xylene (solution replaced every 24 h). Plastic infiltration was performed using methyl methacrylate (MMA, Sigma M55909) for one week at 4 °C. Samples were then embedded in MMA containing 0.5% Perkadox 16 (Dr. Grogg Chemie AG, Stettlen, Switzerland, G425), 15% dibuthylthalate (Sigma-Aldrich, St. Louis, MO, USA, 524980), and 0.01% Pentaerythritol tetrakis (Sigma-Aldrich 441783) for 72 h at 4 °C. Polymerization was continued for 48 h at 26 °C and through subsequent temperature increases over 1 week to 37 °C (bench top incubator with air supply, Memmert, Scharbach, Germany) while constantly checking for polymerization progress. Fully polymerized samples were cut using a Mecatome T180 (Presi, Le Locle, Switzerland) and further processed to 10 µm sections (Leica Reichert Jung Polycut S, Austin, TX, USA). Sections from the middle of the samples were stained using the Goldner’s Trichrome method. Images were acquired using a Leica microscope with a millimeter scale and were analyzed using the Adobe Photoshop program.

### 2.6. Statistical Analysis

All statistical analyses were performed with IBM SPSS statistics 25. Results are expressed as the mean ± SD and were compared by the non-parametric Kruskal–Wallis test followed by the Mann–Whitney U test. Results were considered significantly different for *p* < 0.05.

## 3. Results

### 3.1. Loading of Membranes with DMA

**Figure 1:**
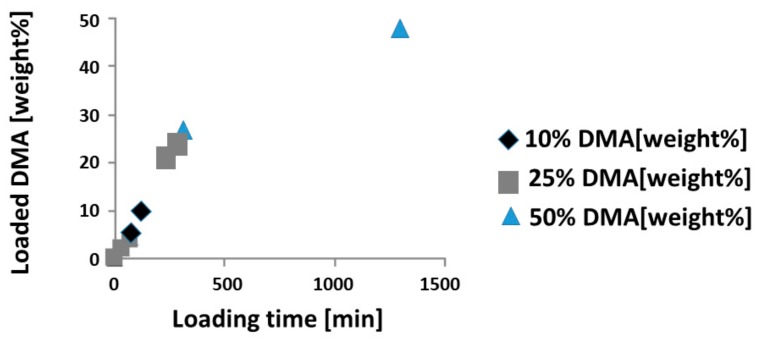
Vapor deposition of *N,N*-Dimethylacetamide (DMA). Vapor deposition up to 22 h is presented. A total of 1320 min are needed to reach 50 wt % DMA loading. Black diamonds indicate loading for the vapor 10% sample, which needs 120 min. The dark grey squares represent the vapor 25% sample, needing 300 min. Light blue triangles depict the loading curve for the vapor 50% sample.

To utilize the PLGA-based guided bone regeneration membrane, we first studied the uptake of DMA via vapor deposition, where a membrane is subjected for different time intervals to DMA vapor. Loading the membrane with 10 wt % DMA is achieved within 2 h. A 25 weight percentage loading needs 5 h, and 50 weight percentage loading 22 h, respectively (Figure 1).

When dipped in pure DMA it takes only 10 s for the uptake of DMA to reach 50 weight percentage. Since DMA is a volatile substance, upon loading the DMA membranes had to be stored in a closed compartment.

### 3.2. DMA Release from Membrane

The storage of the vapor 50%-loaded membranes in air led to a decrease in the DMA-load by 20% of the original loading over a four-day period. The dip-loaded membranes lost 90% of their DMA in the first 5 h after exposure to air. Therefore, the storage and eventual distribution of loaded membranes could cause problems.

**Figure 2:**
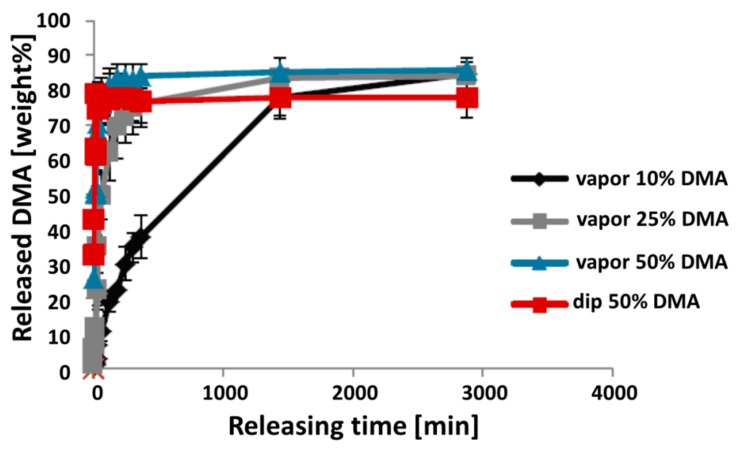
In vitro release of DMA from membranes, within forty-eight hours of measurement. Loading patterns are as follows: black diamond, DMA vapor exposure to 10 wt %; grey squares, vapor to 25%; blue triangle, vapor to 50%; red squares, dip-loaded to 50%. Total release of all the contained chemical into the PBS solution is referred to as 100% release. Individual values for each time point are represented as a percentage of maximal possible release or “cumulative release”.

Next, we performed release studies of loaded membranes in PBS to mimic the in vivo situation. Released DMA was assessed for each time point individually while the collected sample volumes were taken into account (Figure 2). Clear differences between the release kinetics exist between all of the differentially treated membranes. While the vapor 10% membranes released their contained chemical gradually over multiple hours, the dip-loaded membrane released almost all DMA within the first few minutes, reaching a plateau phase at 80% within 30 min. All other membranes reached this plateau phase, which approached the 100% release mark, during the measuring period at four months.

### 3.3. Impact on Membrane Structure

**Figure 3:**
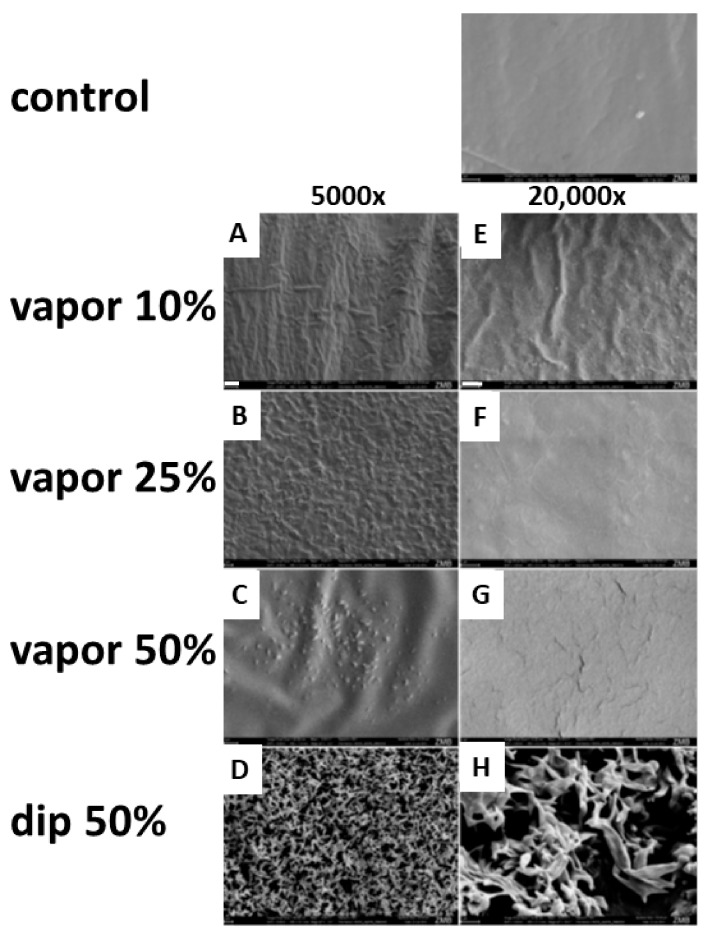
SEM images of native control and DMA-loaded membranes. Control: the surface of an untreated, native membrane is displayed at 20,000× magnification. (**A**–**D**) Images at 5000× magnification (white scale bar in A represents 2 µm for the entire column), (**E**–**H**) images at 20,000× magnification (white scale bar in E represents 1 µm for the entire column).

These release kinetics indicated a difference between the two protocols for loading the membranes. A difference in appearance was observed right after the loading of the chemical. The dip-loaded membrane “dip 50%” (d50) turned opaque and rigid shortly after loading, in contrast to the vapor-loaded membranes, which stayed transparent and flexible after loading with DMA. To further investigate the effect of the different methods of chemical loading onto the structure of the membranes, scanning electron microscopy (SEM) was performed. A non-treated membrane served as a control for the initial membrane surface morphology (Figure 3).

### 3.4. In Vivo Experiment with a DMA-Loaded Membrane

To estimate the expected effective concentration of DMA released from the DMA-loaded membranes into the calvaria defect rabbit model, the size of the applied membranes was set to 2 × 38 mm^2^ and the volume enclosed by the two membranes was set to 100 µL. Based on these estimations, the calculated concentrations for all but the vapor 10% membranes exceeded 10 mM, which is toxic for cells [22]. Therefore, only the membrane vapor 10% was chosen for the in vivo experiment.

**Figure 4:**
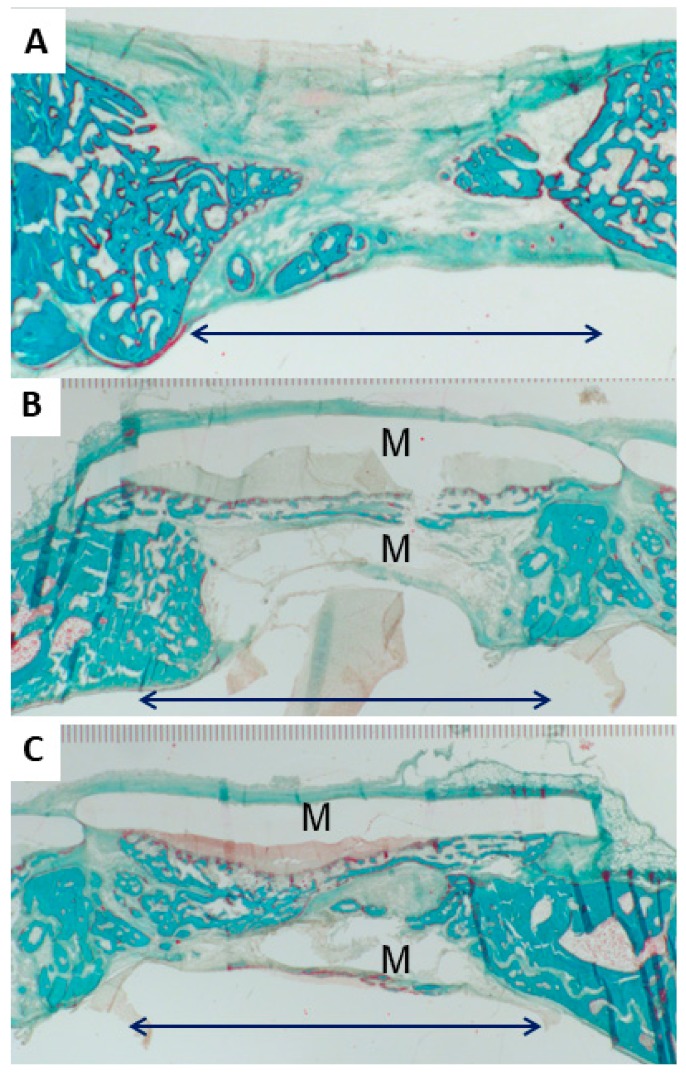
Goldner’s Trichrome staining of calvarial defects treated with poly-lactide-co-glycolide (PLGA)-based membranes (**A**) empty control defect, (**B**) defect treated with two native PLGA membranes (membrane no DMA), (**C**) defect treated with two PLGA membranes loaded to 10 wt % with DMA (membrane vapor 10%). M marks the transparent layer of the membranes placed above and below the defect. Black arrows indicate the defect width of six mm. Trichrome stains the bone in a greenish-blue color and the osteoid in red.

After operation, the animals behaved normally, suggesting that the choice of the membrane vapor 10% and the released DMA had no negative effects on the well-being of the rabbits. Bone regeneration was allowed for four weeks. After sacrifice, calvarias were collected and processed for histological analysis. Examples of the histologies of the different treatments are presented in Figure 4.

**Figure 5:**
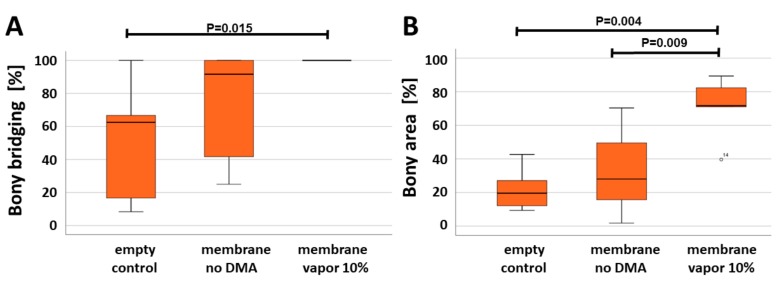
Analysis of bone regeneration parameters. (**A**) Quantification of bony bridging represented as a percentage of the 6 mm defect, (**B**) bony regenerated area as a percentage of pixel numbers identified as bone in the trichrome staining in relation to the entire defect. Empty refers to the defects without any membrane. Membrane no DMA refers to the native PLGA membranes. Membrane vapor 10% refers to the PLGA membranes pre-loaded with 10 wt % DMA via vapor deposition. Values are displayed as box plots ranging from the 25th (lower quartile) to the 75th (upper quartile) percentile, including the median as a solid black line and whiskers showing the minimum and maximum values. In (B) values outside the range of the box plot are shown as individual points. (*p* values are provided).

The histomorphometric analysis (Figure 5), based solely on the middle section from the defect, showed that the bony bridging achieved by the application of the vapor 10% membrane (100.00 ± 0.00%) was significantly higher than compared to the empty control defect (52.78 ± 34.49%) (*p* < 0.015). No significant difference was seen with the native PLGA membrane (membrane no DMA) (75.00 ± 33.33%), neither compared with the empty control defect or with the vapor 10% DMA-loaded membrane (membrane vapor 10%). The bony regenerated area achieved with the vapor 10% DMA-loaded membrane (membrane vapor 10%) (70.93 ± 17.04%) was, however, significantly higher than the empty control defect (21.71 ± 12.22%), and the defect treated with unloaded membrane (membrane no DMA) (32.21 ± 24.51%).

## 4. Discussion

The evolution of guided bone regeneration membranes has gone from barrier membranes to biodegradable barrier membranes to bioactive biodegradable barrier membranes [9]. Here we characterize a guided bone regeneration membrane of the third generation, which serves as barrier, is biodegradable, and is bioactive due to the release of DMA.

DMA is an FDA-approved excipient and is used as a solvent in diverse formulations of drugs [19,24]. Despite its clinical use as an excipient, its diverse activities have been overlooked for many years [21]. More recently, we have identified the underlying mechanism for all these diverse and beneficial activities. DMA binds bromodomains, based on the low affinity of DMA for this acetyl-residue-binding site [22]. Since bromodomains are an important element to assemble the machinery for NF-kappa-B transcription [25], DMA significantly attenuates lipopolysaccharide- and tumor necrosis factor-induced proinflammatory responses [26]. In conjunction with guided bone regeneration, a reduction in inflammation could be beneficial, and so would the enhancement of the activity of bone morphogenetic protein signaling, and the inhibition of osteoclast differentiation and activity [22]. This notion proved true in our in vivo experiments, where the DMA-loaded membrane performed significantly better in terms of bony bridging than the control (Figure 5). Even more striking was the performance of the DMA-loaded membrane in the extent of bony regeneration for the defect area. In this aspect, the DMA-loaded membrane performed significantly better than the native membrane and the control. Therefore, local and sustained release of DMA is beneficial in conjunction with guided bone regeneration procedures. Nevertheless, more studies in larger animals, in alveolar bone models, and with additional time points are needed to further substantiate this finding.

The major problem utilizing DMA as a drug is achieving a sustained delivery of dosages in the millimolar range, since its affinity to bromodomains is in this range [22]. Therefore, we tried the vapor deposition methodology, which we have used before to load membranes with N-methyl pyrrolidone (NMP) [27], another FDA-approved excipient. In essence, we achieved very similar results to what we did with NMP and selected the vapor 10% loaded membrane for the in vivo test. The two reasons were, firstly, that the vapor 10% membrane provides the most sustained DMA delivery in a non-toxic range (Figure 2). Secondly, the structure and integrity of the membrane was preserved the most with this loading regime (Figure 3).

By combining a PLGA membrane with DMA, we developed another guided bone regeneration membrane of the third generation. DMA added bioactivity to the system, as evidenced by the increased bony regeneration and bony bridging (Figure 5). In the direct comparison of bony regeneration vapor 10% NMP, another FDA-approved excipient reached 67.10 ± 10.54% [1] and vapor 10% DMA (70.93 ± 17.04%). For bony bridging, vapor 10% NMP reached 89.24 ± 14.34% and vapor 10% DMA 100.00 ± 0.00%. Both excipients, NMP and DMA, appear to have a similar potency in terms of bone regeneration and bony bridging, but the lower volatility of NMP makes it more suitable for carrier systems like the PLGA membrane. For systemic applications via injections, however, DMA has been used very successfully in clinical and preclinical trials [19,21,22,28].

More advanced in the field of guided bone regeneration research is the application of NMP. For the NMP-loaded membrane, the dip-loaded version was used for preclinical [11,29] and two clinical trials [16,17]. Unfortunately, in both clinical trials the dip-loaded NMP-releasing membrane was only equal to (but failed to outperform) clinically well-established membranes. Whether a membrane with NMP deposited by vapor could lead to better results in humans has not been tested yet. In a preclinical trial, however, the vapor 10% NMP performed significantly better than the dip-loaded NMP membrane [27]. Furthermore, calcium phosphate-based bone substitutes have been tested as delivery systems for NMP. However, in the preclinical sinus lift model, no benefits of NMP could be detected [18].

## 5. Conclusions

The local delivery of DMA in conjunction with a PLGA-based guided bone regeneration membrane significantly enhanced bony regeneration and bony bridging, thus adding bioactivity to the system, and turning it into a third-generation membrane. However, more research with additional and more relevant animal models is needed to develop the ideal delivery system to maintain the relatively high concentrations of DMA needed to exert its bioactivities efficiently over a longer period.

## 6. Patents

The University holds a patent on DMA in conjunction with osteoporosis.
